# The Role of Recombinant AAV in Precise Genome Editing

**DOI:** 10.3389/fgeed.2021.799722

**Published:** 2022-01-13

**Authors:** Swati Bijlani, Ka Ming Pang, Venkatesh Sivanandam, Amanpreet Singh, Saswati Chatterjee

**Affiliations:** Department of Surgery, Beckman Research Institute, City of Hope National Medical Center, Duarte, CA, United States

**Keywords:** AAV, genome editing, homologous recombination, gene therapy, genetic diseases, rare diseases

## Abstract

The replication-defective, non-pathogenic, nearly ubiquitous single-stranded adeno-associated viruses (AAVs) have gained importance since their discovery about 50 years ago. Their unique life cycle and virus-cell interactions have led to the development of recombinant AAVs as ideal genetic medicine tools that have evolved into effective commercialized gene therapies. A distinctive property of AAVs is their ability to edit the genome precisely. In contrast to all current genome editing platforms, AAV exclusively utilizes the high-fidelity homologous recombination (HR) pathway and does not require exogenous nucleases for prior cleavage of genomic DNA. Together, this leads to a highly precise editing outcome that preserves genomic integrity without incorporation of indel mutations or viral sequences at the target site while also obviating the possibility of off-target genotoxicity. The stem cell-derived AAV (AAVHSCs) were found to mediate precise and efficient HR with high on-target accuracy and at high efficiencies. AAVHSC editing occurs efficiently in post-mitotic cells and tissues *in vivo*. Additionally, AAV also has the advantage of an intrinsic delivery mechanism. Thus, this distinctive genome editing platform holds tremendous promise for the correction of disease-associated mutations without adding to the mutational burden. This review will focus on the unique properties of direct AAV-mediated genome editing and their potential mechanisms of action.

## Introduction

The completion of the sequencing of the human genome marked the start of the race to create targeted modifications for the study of gene function, generation of disease models, and therapeutic applications (Li et al., 2020; Galichet and Lovell-Badge, 2021). Subsequent extensive genome-wide association studies linked defined genetic mutations in the population with known diseases. However, despite intensive efforts in these areas, few curative genetic therapies have emerged to date. It is estimated that currently, less than 5% of rare genetic diseases have effective treatments (Brooks et al., 2020). Protein replacement therapies have been developed for many inherited diseases. However, the availability, cost, and quality of life issues pose significant barriers. Moreover, the inability of many recombinant proteins to cross the blood-brain barrier and their immunogenicity in patients with null mutations pose additional therapeutic challenges. Thus, the ability to precisely and permanently correct pathogenic mutations at the level of the genome without adding to the mutational burden has the potential to be transformative for genetic therapies of inherited and acquired diseases. Here, we will review the unique contributions of adeno-associated virus (AAV) vectors to the field of genome editing in the larger context of genetic medicine.

## Adeno-Associated Viruses

**FIGURE 1:**
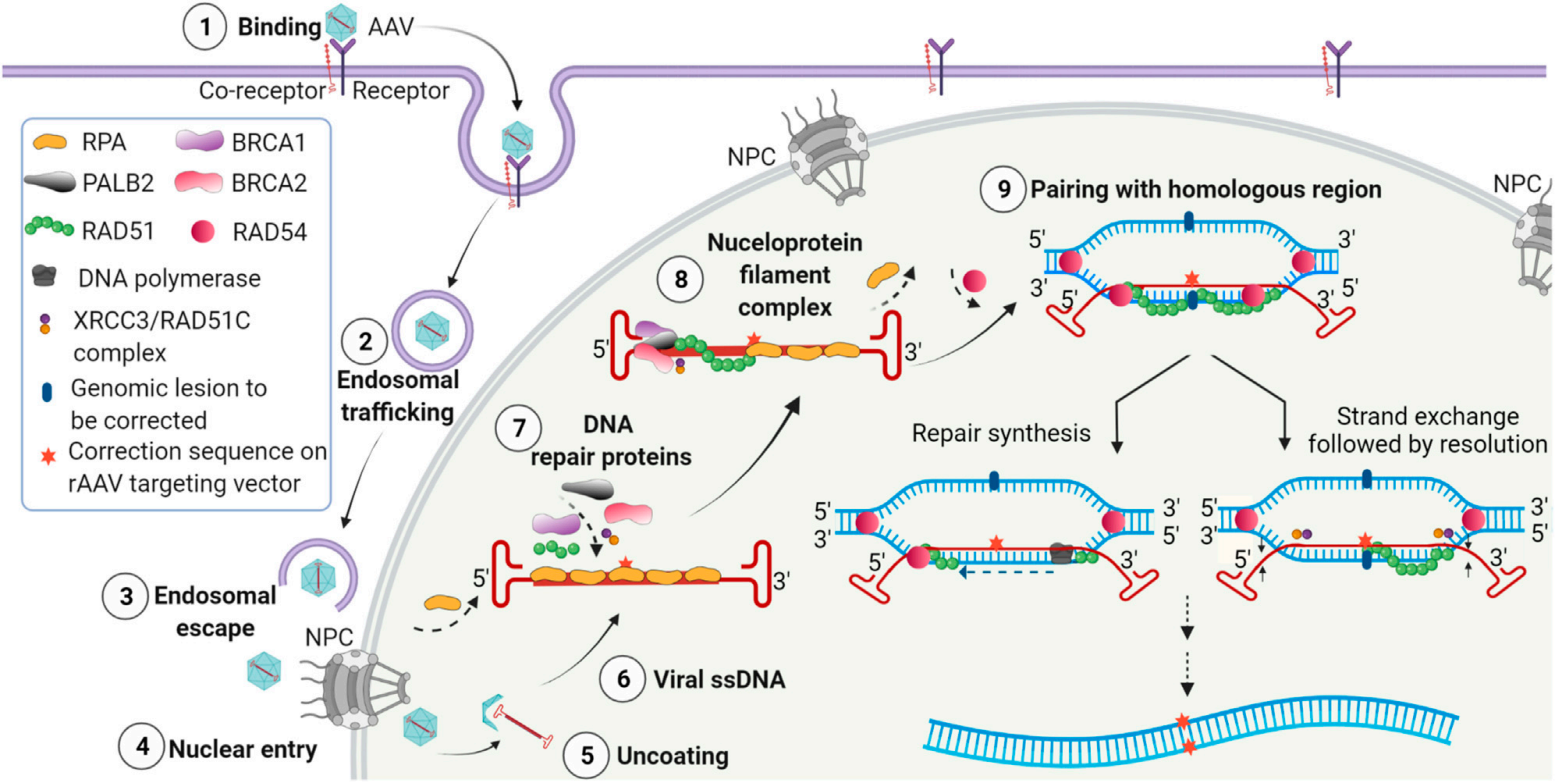
Model of events following transduction by AAV editing vectors leading to genome editing. **(1)** Binding of AAV virions to cell surface receptor and co-receptors and initiation of receptor-mediated endocytosis. **(2)** Endosomal entry and trafficking of the AAV virions. **(3)** Release of AAVs from endosomes. **(4)** Entry of AAV through the nuclear pore complex (NPC). **(5)** Uncoating of AAV virions and release of the single-stranded vector genome in the nucleus. **(6)** The released single stranded AAV vector genome with homology region and ITRs. **(7)** Recruitment of DNA repair proteins to the AAV editing genome containing the correction sequence. **(8)** Assembly of the HR complex on the AAV editing genome and formation of nucleoprotein filament complex. **(9)** Homology search between editing vector genome and chromosomal DNA leading to the pairing of homologous sequences and initiation of editing by repair synthesis (left) or strand exchange (right).

**FIGURE 2:**
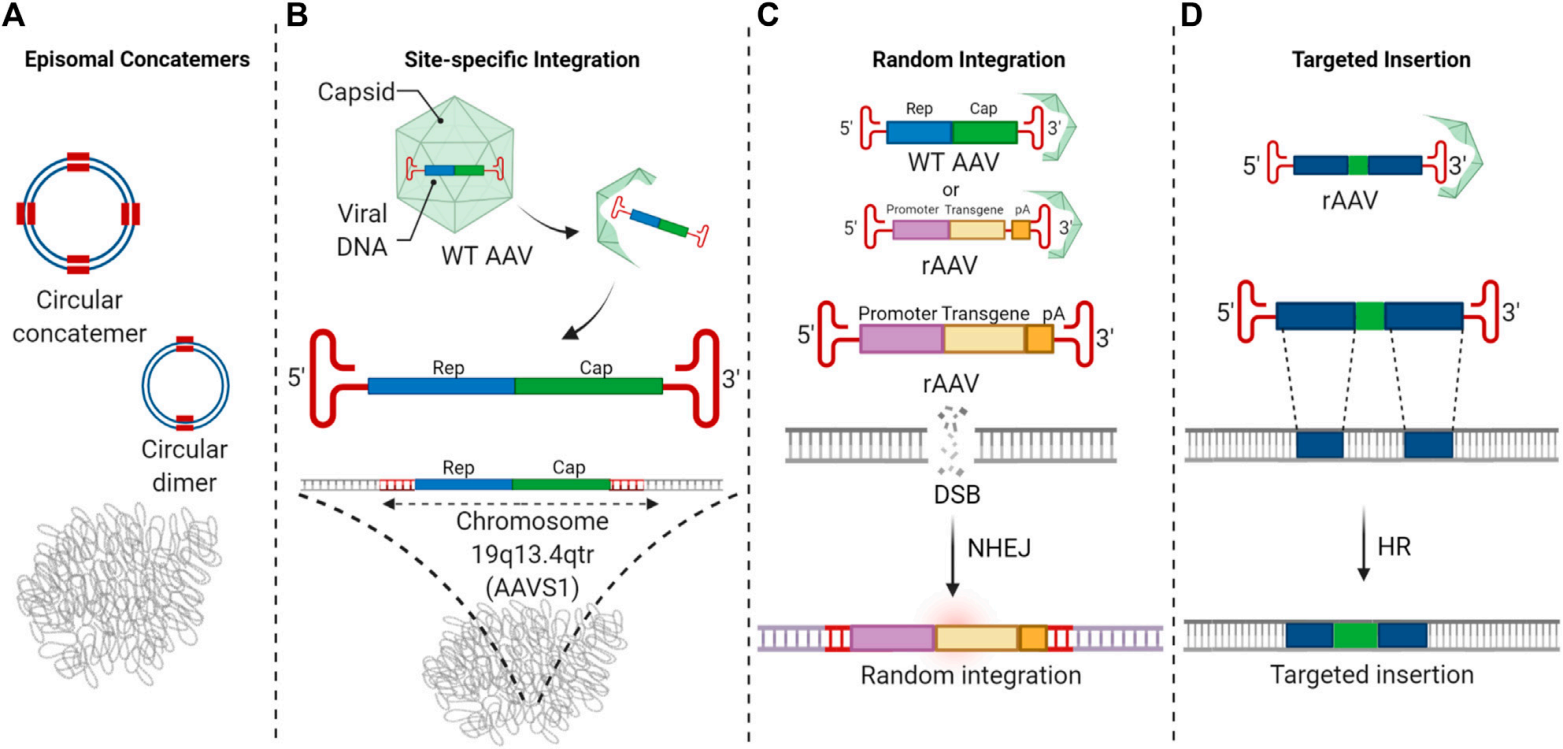
The fate of AAV vector genomes in the nucleus. **(A)** AAV vector genomes predominantly survive long-term as episomal concatemers. **(B)** Wild-type AAV and Rep 68/78 containing AAV vectors can undergo site-specific integration at the AAVS1. **(C)** A small fraction of AAV vector genomes undergo random integration at very low frequencies. This event involves the AAV ITRs. **(D)** HR-mediated AAV editing results in targeted insertion at chromosomal locations specified by the homology arms.

AAVs have emerged as efficient genetic modification vehicles due to efficient *in vivo* infectivity, non-pathogenicity, widespread tissue tropism, rare genomic integration, and their ability to infect and persist in non-dividing cells (Gaj et al., 2016; Epstein and Schaffer, 2017). AAVs are comprised of a family of natural human non-pathogenic, single-stranded, replication-defective parvoviruses (Berns and Linden, 1995; Srivastava, 2016). The single-stranded AAV genomes are bounded at either end by palindromic G-C-rich inverted terminal repeats (ITRs), which self-base-pair to form unique structures. AAV infection of target cells in the absence of helper virus coinfection results in latency which is the basis for the use of AAV as delivery vehicles for genetic material. AAV infection of target cells is initiated by binding to a receptor and/or a co-receptor (Figure 1). AAV virions are then internalized and translocated to the nucleus via an endosomal route and enter the nuclear pore complex (Junod et al., 2021). AAV virions then undergo uncoating in the nucleus, and the single-stranded genomes are released. AAV genomes localize to the periphery of the nuclei and colocalize with euchromatin, where active transcription and DNA repair are known to occur. For wild-type AAV, if a helper virus is present, replication ensues. AAV replication utilizes the AAV encoded Rep proteins, helper virus-encoded functions, and the cellular replication proteins RPA, RFC, PCNA, and DNA polymerase delta (Ni et al., 1998; Nash et al., 2007). Recombinant AAV vectors are doubly replication-deficient in the absence of AAV Rep/Cap genes and helper virus functions and do not undergo replication. After uncoating, the single-stranded genomes are converted to double-stranded multimeric circular concatemeric episomal forms that persist long-term in post-mitotic cells (Figure 2A) (Ferrari et al., 1996; Wang et al., 2007).

Recombinant AAV vectors have proven to be safe, well-tolerated, and effective gene therapy vectors for treating genetic diseases. Over 3,300 individuals have been treated with AAV vectors, and there are over 130 AAV trials registered on clinicaltrials.gov (Kuzmin et al., 2021). Two AAV vectors have received USFDA approval, Luxturna for a rare inherited retinal dystrophy and Zolgensma for spinal muscular atrophy. AAV vectors are primarily safe, with a few exceptions resulting from very high-dose treatments in specific disease settings (Kuzmin et al., 2021). It is expected that with improvements in vector design, production, and purification methods, the toxicities associated with high-dose treatment will be controlled. Thus, AAV vectors are well on their way to becoming established genetic therapies.

## Limitations of Gene Therapy

However, despite the tremendous promise of gene therapy to cure genetic diseases, several limitations remain. First, since AAV vector genomes primarily exist as nuclear episomes, the long-term durability of treatment is a concern. It is unclear whether the episomal AAV vector genomes will persist for the life of the patient. Since most cells in the adults are post-mitotic, it is likely that gene therapy will last for several years, as has been documented in many trials (Niemeyer et al., 2009; Nathwani et al., 2011; Nathwani et al., 2014). However, the consequences of cellular turnover in adult tissues on the persistence of episomal AAV are yet to be defined. Similarly, the long-term fate of AAV gene therapy in infants and children whose tissues are actively undergoing growth and expansion will become clear over time.

Secondly, while gene therapy is ideally suited for the treatment of autosomal and X-linked recessive diseases, where expression of a transgene that encodes a missing protein is sufficient to overcome the deficiency associated with the disease, treatment of autosomal dominant disorders is more challenging. For example, treatment with an AAV vector encoding the clotting factor VIII overcomes the deficiency in the X-linked recessive disease, hemophilia A. However, for autosomal dominant disorders, expression of mutant proteins may have a dominant-negative effect or cause toxicity, such as in Huntington’s disease. In such cases, strategies for allele-specific silencing of expression are necessitated but challenging to achieve with gene therapy.

Lastly, while gene therapy in its current iteration readily addresses diseases where the expression level is not critical, it is much harder to use to treat conditions where the tolerated window of transgene expression is narrow. In these cases, often, either too little or too much transgene expression leads to toxicity. An example of this is Rett syndrome, caused by mutations in the MECP2 gene (Amir et al., 1999). MECP2 expression is highly regulated *in vivo*, and either too much or too little expression leads to toxicity (Montgomery et al., 2018; D'Mello, 2021). Most gene therapy vectors utilize heterologous promoters since the natural chromosomal promoters of most disease-associated genes exceed the coding capacity of AAV vectors. Additionally, regulatory sequences for many genes have yet to be identified. Even when identified, the addition of regulatory sequences to AAV gene therapy vectors may again be limited by the coding capacity of AAV. Thus, achieving physiologic regulation of transgene expression from a gene therapy vector is challenging. Hence, despite the tremendous achievement of AAV gene therapy in curing previously incurable genetic diseases, other approaches are necessary to address these recognized limitations.

One solution is to repair pathogenic mutations precisely and accurately at the level of the genome such that the correction would last for life and natural physiologic gene regulation would be maintained. This is enabled by genome editing technologies.

## Programmable Nuclease-Based Genome Editing Platforms

A key breakthrough in genome editing was the observation that editing could be enhanced by homologous recombination (HR) (Capecchi, 1989) in human cells and *in vivo* (Smithies et al., 1985; Thomas and Capecchi, 1987). However, the frequency of HR usually is very low, ∼1 event in 10^6^–10^9^ cells, rendering it challenging for therapeutic applications (Capecchi, 1989). A key advance in genome modification was the observation that the creation of double-stranded DNA breaks (DSBs) at targeted sites could enhance editing through the use of HR (Rudin et al., 1989; Plessis et al., 1992; Choulika et al., 1995; Bibikova et al., 2001; Bibikova et al., 2003). This spurred the development of editing platforms based upon programmable nucleases to induce targeted DSBs. In the presence of correction DNA templates bearing homology to the target genomic sequence, a fraction of DSBs were found to undergo HR-based editing (Vasileva et al., 2006; Wang et al., 2016; Smith et al., 2018), resulting in a recent proliferation of programmable nuclease-based editing platforms. These include meganucleases (Stoddard, 2005; Smith et al., 2006), zinc finger nuclease (ZFNs) (Porteus and Baltimore, 2003; Urnov et al., 2005; Wood et al., 2011), TALENs (Boch et al., 2009; Christian et al., 2010; Wood et al., 2011; Zhang et al., 2011) and CRISPR/Cas9 (Horvath and Barrangou, 2010; Gasiunas et al., 2012; Jinek et al., 2012; Cong et al., 2013; Mali et al., 2013b). The CRISPR/Cas9 platform has recently been further refined to replace DSBs with single-stranded nicks resulting in the base editing and prime editing platforms (Anzalone et al., 2019; Matsoukas, 2020). However, it was shown that in addition to the high-fidelity homology dependent repair (HDR), DSBs could also be repaired by the Non-Homologous End Joining (NHEJ) pathway (Chapman et al., 2012). The NHEJ pathway, which is thought to have developed as a stop-gap repair mechanism to patch DNA breaks in the mid-cell cycle, is error-prone as it is carried out without a homologous repair template. DNA repair by NHEJ is often associated with insertion/deletion (indels) mutations. While HR, which primarily occurs during mitosis and utilizes sister chromatids as repair templates, is error-free and precise and geared towards maintaining genomic integrity. These DNA repair pathways are being leveraged for gene editing to enable the versatile and accurate correction of pathogenic mutations. The major genome editing platforms based upon programmable nucleases are briefly reviewed below.

### Meganucleases

Meganucleases are sequence-specific endonucleases that recognize 15–30 bp cleavage sites. Meganucleases such as homing endonucleases I-SceI and I-CreI (Hoess et al., 1982; Monteilhet et al., 1990; Hasan et al., 1994; Stoddard, 2005; Smith et al., 2006) have relatively high specificity and precision. Because of their relatively long recognition sequence, target sites are rare in any genome. Strategies have been developed to retarget them to novel sequences, thus expanding their use for genome editing (Smith et al., 2006; Silva et al., 2011). However, tailoring meganucleases is laborious and requires significant protein engineering (Smith et al., 2006). In addition, the lack of defined DNA binding and cleavage domains further hampers protein engineering efforts, rendering large-scale use challenging.

### Zinc Finger Nucleases (ZFNs)

ZFNs are artificially engineered fusion proteins that consist of zinc finger DNA-binding domains and a DNA-cleavage domain (Bibikova et al., 2003; Porteus and Baltimore, 2003; Porteus and Carroll, 2005; Urnov et al., 2005; Miller et al., 2007; Wood et al., 2011). Each zinc finger is relatively small at about 30 amino acids and can bind a three base pair DNA sequence. Tandem zinc fingers are required to create sequence specificity to target a given locus. Complex and expensive protein engineering is necessary to achieve sequence specificity. However, high levels of affinity and specificity of the system are difficult to achieve, resulting in a high frequency of off-target cleavage.

### Transcription Activator-Like Effector Nucleases (TALENs)

TALENs are also engineered fusion proteins comprised of tandem arrays of 10–30 DNA recognition repeats and the FokI endonuclease (Boch et al., 2009; Moscou and Bogdanove, 2009; Christian et al., 2010; Cermak et al., 2011; Mahfouz et al., 2011; Miller et al., 2011; Wood et al., 2011; Zhang et al., 2011; Reyon et al., 2012). The repeats are derived from transcription activator-like effectors (TALEs) that contain 33–35 amino acids with two adjacent amino acids termed the repeat-variable di-residue (RVD). The RVDs confer binding specificity to one of the four DNA base pairs. Thus, engineering DNA binding domains to specific sequences can be achieved by stitching different repeats together. Although the process is straightforward, the construction of each TALEN array is time-consuming and labor-intensive, limiting their use in high-throughput applications.

### Clustered Regularly Interspaced Short Palindromic Repeats (CRISPR/Cas9)

The CRISPR/Cas9 system is one of the later additions to the toolbox of programmable nuclease-based genome editing (Haurwitz et al., 2010; Deltcheva et al., 2011; Gasiunas et al., 2012; Jinek et al., 2012; Cong et al., 2013; Mali et al., 2013b). It is built upon the adaptive immunity of bacteria and archaea, whereby the foreign DNA sequences (protospacers) of bacteriophage and plasmids are integrated into copies of repeat sequences named CRISPR (Jansen et al., 2002), which serve as marks of memory of previous attackers and act as a surveillance defense mechanism. Short guide RNAs transcribed from the protospacers form complexes with Cas nucleases to search and destroy incoming matching foreign DNA (Haft et al., 2005; Makarova et al., 2006). The more straightforward class II CRISPR system that consists of a single guide RNA (sgRNA) and Cas9 protein has been widely adapted for gene manipulations. The sgRNA consists of a scaffold sequence for binding to Cas9 and a 20 base pair spacer for sequence recognition that guides Cas9 to the cleavage target (Sapranauskas et al., 2011; Chylinski et al., 2013). The Cas9 nuclease recognizes a protospacer-adjacent motif (PAM) (Mojica et al., 2009) of 3′-NGG and cleaves 3-4 base pairs upstream of the PAM sequence. Since NGG motifs are abundant in most genomes, the CRISPR/Cas9 system offers flexible targeted cleavages at numerous genomic loci. By simply changing the protospacer sequence, any sequence in the genome could be potentially targeted and manipulated, making it ideal for high-throughput applications. These properties also allow easy adaptation for multiplexing by simply introducing multiple guide RNAs into the same cell to achieve multiple manipulations simultaneously (Cong et al., 2013). In addition, engineered Cas9 and the discovery of other Cas proteins that recognize different PAM sites further expand the flexibility of this platform. To avoid detrimental mutations associated with DSBs, new functions other than DNA cleavage have been engineered into Cas9 to mediate processes such as nicking (Mali et al., 2013a; Ran et al., 2013), gene activation (Cheng et al., 2013; Perez-Pinera et al., 2013; Qi et al., 2013; Konermann et al., 2015), gene suppression, (Bikard et al., 2013) directed base editing, and prime editing (Komor et al., 2016; Nishida et al., 2016). The latter two strategies that allow genome editing without donor DNA are discussed further below.

### Base Editing

Directed base editing and prime editing allow genome editing without the requirement for DSBs or a donor template. Base editing utilizes a nuclease defective Cas9 mutant (dCas9) (Jinek et al., 2012) fused to a cytidine deaminase. The fusion protein then targets a genomic locus where the cytidine deaminase converts any C to U within a five base-pair window, thereby directly mediating a C to T (G to A on opposite strand) conversion (Komor et al., 2016). To broaden the scope of this platform, an engineered RNA deaminase was developed to convert the A-T base pair to a G-C base pair (Gaudelli et al., 2017). To further improve versatility and specificity, extensive engineering was also carried out on dCas9 to recognize different PAM sites and on the cytidine deaminase to narrow the targeting window from approximately 5 to 1-2 base pairs (Kim et al., 2017). However, the direct base editing strategy is limited to converting single bases but not for correction of insertion or deletion mutants.

### Prime Editing

The prime editing platform may address this limitation of base editing (Anzalone et al., 2019; Anzalone et al., 2020). The key components of prime editing are the prime editing guide RNA (pegRNA) and the Cas9 nickase-reverse transcriptase (RT) fusion protein. pegRNAs contain extra sequences at the 3′ end of the sgRNA that act as a priming site for the nicked DNA and serve as a template for reverse transcription. Target search is mediated by the spacer sequence of the pegRNA. Upon binding to the target strand, the displaced DNA strand, R-loop DNA, is nicked by Cas9 nickase (Ran et al., 2013). The 3′ end of the nicked DNA can then anneal to the prime RNA sequence and extend to copy the correction sequences by RT. The corrected ssDNA flap can then anneal back to the target strand, replace the 5′ flap and result in a heteroduplex with one copy of the corrected sequence. Further nicking of the unedited strand can result in the correction of both strands. Both direct editing and prime editing avoid double-strand DNA breaks, therefore, dramatically lowering DSB-induced on-target indels and, more critically, extensive scale gene rearrangement. However, the high frequency of off-target recognition remains the primary concern for therapeutic applications (Pattanayak et al., 2013; Tsai et al., 2015). Among the 20 base pair spacer sequences that confer specificity, only the 8–10 base seed sequence (Semenova et al., 2011; Wiedenheft et al., 2011) at the 3′ end is most stringent while the rest allows a certain degree of mismatch, resulting in off-target recognition.

## Limitations of Nuclease-Based Editing Platforms

All programmable nuclease-based platforms require the generation of DSBs or single-stranded nicks designed to enhance HR. However, each platform has certain limitations. Nuclease-mediated genomic DSBs undergo repair mostly via NHEJ, which occurs more frequently than HDR. Since NHEJ is an error-prone repair pathway, indel mutations are often observed at the target sites. These mutations span 1 to 10 base pairs and may result in frameshifts, leading to nonfunctional or mutated proteins. Indel mutations can be especially deleterious in essential genes or genes involved in tumor suppression. Evaluation of the mutation spectra associated with nuclease-based editing platforms shows that deletions are common with the TALENs platform while larger deletions are associated with ZFNs and CRISPR/Cas9 (Gabriel et al., 2011; Pattanayak et al., 2011). Occasional substitutions, inversions, duplications, and longer insertions or deletions of up to several hundred bases have also been observed, albeit at low frequencies (Fu et al., 2013; Kuscu et al., 2014; Tsai et al., 2015). Even in the presence of donor constructs bearing homology to the target sites, repair by NHEJ was observed more frequently than the higher fidelity HDR, likely because the NHEJ pathway was dominant and active at all stages of the cell cycle (Mukherjee et al., 2019). However, HDR could exhibit higher frequency than NHEJ in the presence of very high copy number of donor DNA or when NHEJ pathway genes were knocked down (Certo et al., 2011). Multiple efforts are underway to increase the frequency of HDR after DSB creation, including the use of small-molecule inhibitors of the NHEJ pathway.

Off-target cleavage is likely the most significant concern for programmable nuclease-based editing platforms since the DNA recognition specificity of the nucleases is not precise. This poses a considerable challenge due to the risk of mutagenesis throughout the genome. Promiscuous cutting by programmable nucleases at off-target sites in the genome has been extensively documented (Gabriel et al., 2011; Pattanayak et al., 2011; Fu et al., 2013; Pattanayak et al., 2013; Duan et al., 2014; Kuscu et al., 2014; Lin et al., 2014; Wang X. et al., 2015). DSBs created by the off-target action of programmable nucleases are also repaired via the error-prone NHEJ pathway, adding to the genomic mutational burden. Excessive generation of off-target DSBs has been shown to be cytotoxic (Porteus, 2006; Miller et al., 2007; Szczepek et al., 2007; Guo et al., 2010; Doyon et al., 2011). Improvements by in silico design have led to a reduction but not prevention of off-target cleavage. Significant off-target cleavage has been reported in these platforms, including CRISPR/Cas9. Off-target cleavage by Cas9 with up to 5 base pair mismatches between the target DNA and the guide RNA has been documented. Previous studies have shown that the different structures of the guide RNA can influence the cleavage of on-target and off-target sites, thereby raising concerns over their use.

Most nuclease-based editing platforms comprise of multiple components. The precise delivery of each element to the nuclei of target cells is critical for genome editing. While *in vitro* or *ex vivo* applications can be achieved by viral vector-mediated delivery or direct transfection of plasmid DNA or synthetic mRNA, *in vivo* delivery to target tissues remains challenging. Recently, AAVs have become the vector of choice for delivering programmable nucleases and the donor template for HDR or both (Wang et al., 2015; Sather et al., 2015; Dever et al., 2016; Wang et al., 2016). However, the limited coding capacity (∼4.8 kb) of AAV vectors remains a hurdle for delivering nuclease-based editing components.

## AAV Vectors for Genome Editing

All AAVs possess unique inherent genome editing properties that set them apart from all other editing platforms. They utilize a high-fidelity repair pathway that does not introduce additional mutations during the editing process and do not require the use of exogenous nucleases to create DSBs before editing. AAVs edit the cellular genome independently of the cell cycle stage and equally well *in vitro* and *in vivo*. Here, we will focus on the intrinsic and distinctive genome editing properties of AAV vectors and the potential mechanisms by which they function. It has long been known that AAV vectors mediate targeted insertion in the cellular genome (Russell and Hirata, 1998; Inoue et al., 1999; Miller et al., 2003; Porteus et al., 2003; Vasileva et al., 2006; Khan et al., 2011; Barzel et al., 2015; Smith et al., 2018). Early studies showed that gene targeting efficiency with AAV2 was approximately 2-3 logs higher than other methods (Russell and Hirata, 1998). Gene targeting in human cell lines was shown to occur at frequencies of approximately 0.1–1% (Russell and Hirata, 1998; Porteus et al., 2003). AAV gene targeting has been demonstrated at multiple genomic locations, including genes such as hypoxanthine phosphoribosyltransferase (HPRT), Type I collagen (COL1A1) (Hirata et al., 2002; Chamberlain et al., 2008), human and murine IL2RG (Hiramoto et al., 2018; Smith et al., 2018), the AAVS1 safe harbor locus (Smith et al., 2018), human phenylalanine hydroxylase (PAH) (Chen et al., 2020), and the murine ROSA26 locus (Miller et al., 2006; Smith et al., 2018). Recombinant AAV was used to site-specifically target therapeutic transgenes to the 3′ untranslated region of the albumin gene, such that expression was driven by the albumin promoter. Transgenes were coexpressed with a short hairpin RNA. Coupling this approach with targeted expression from the albumin promoter led to supraphysiologic levels of Factor IX expression (Barzel et al., 2015; Nygaard et al., 2016). Using a similar approach, expression of methylmalonyl-CoA mutase from the albumin promoter was shown to have efficacy in a murine model of methylmalonic acidemia (Chandler et al., 2021). In addition to targeted genomic insertions at specified locations, AAV vectors have been shown to mediate nucleotide substitutions (Smith et al., 2018) and small deletions.

## Genomic Integration of AAV

While intracellular AAV vector genomes predominantly survive as episomes long-term (Figure 2A), in some instances, chromosomal integration of AAVs has been observed. There are two specific forms of AAV integration that are distinct from AAV-mediated genome editing. To distinguish between AAV integration and AAV editing, we will briefly review the forms of AAV integration.

### Site-Specific Integration of Wild-Type AAV

In the absence of helper virus infection, preferential integration of wild-type (WT) AAV genomes was shown to occur at a specific genomic site on chromosome 19q13.2-13.4qtr, also known as the AAVS1 locus (Figure 2B) (Kotin and Berns, 1989; Kotin et al., 1990; Kotin et al., 1991; Samulski et al., 1991; Kotin et al., 1992). Thirty to seventy percent of integrated WT AAV is found at the AAVS1 locus. Site-specific integration by WT AAV requires the presence of the AAV-encoded Rep 68/78 proteins which bind to both the Rep binding site (RBS) element on AAVS1 and on the AAV genome (Linden et al., 1996; Hamilton et al., 2004). Following the formation of a double-stranded intermediate of the WT AAV genome, the AAV p5 promoter is activated to express the Rep proteins. Rep68/78 proteins then mediate complex formation between the AAV genome and AAVS1 locus and generate a nick at the terminal resolution site (TRS) in AAVS1, resulting in the integration of the AAV genome at the AAVS1 locus via a strand switch mechanism. The p5 Integration efficiency element (p5IEE) in the p5 promoter was shown to be required in cis to mediate integration (Philpott et al., 2002a; Philpott et al., 2002b). This element has been reported to also act as an origin of replication (Wang and Srivastava, 1997) which might recruit the replication machinery for strand switching. However, little is known about the host cell factors that are involved in Rep-mediated site-specific integration. Since site-specific integration by AAV at the AAVS1 locus is not associated with pathogenicity or toxicity, it is widely used as a safe harbor locus to insert reporter or therapeutic sequences (Dreyer et al., 2015; Stellon et al., 2021).

### Random Integration of AAV Vectors

In addition to site-specific integration by Rep-containing AAV, Rep-free AAV vectors have been found to undergo random integration at very low frequencies (Figure 2C) (McCarty et al., 2004). Random integration of recombinant AAV in the host genome has been shown to involve the ITRs and potentially regions of microhomology with the genome, possibly indicating a role for the hairpin structure of ITR in NHEJ-mediated integration (Rutledge and Russell, 1997; Yang et al., 1997; Nakai et al., 1999). In these integration events, complete, partial, or rearranged ITR sequences are found at the junctions between chromosomal sequences and AAV vector genomes. Induction of DSBs was shown to result in a higher frequency of integrations, confirming the hypothesis that random integration of AAV is mediated via NHEJ in the presence of DSBs. However, in the absence of DSBs, AAV vectors integrate at very low frequencies, possibly at sites of spontaneous DNA breaks (Miller et al., 2004). AAV vectors have been shown to integrate at regions of genomic instability, for instance, satellite DNA sequences, palindromic sequences, rRNA encoding DNA repeats, and CpG islands. These regions may be more prone to spontaneous breaks resulting in deletions, insertions, and translocations (Nakai et al., 2003; Miller et al., 2005; Nakai et al., 2005; Inagaki et al., 2007; Nguyen et al., 2021). Random integration of AAV vectors into the cellular genome is a rare event that has not been associated with pathology in clinical trials to date, and AAV vectors are considered safe (Gaudet et al., 2013; Nathwani et al., 2014).

### ITR Insertion Into the Chromosome

Two studies have extensively evaluated the sequence of edited chromosomal loci after AAV-mediated HR (Smith et al., 2018; Chen et al., 2020). Evaluation of numerous edited chromosomal sequences by both Sanger and NGS sequencing showed a clear absence of ITR insertions in both cases, indicating that ITRs are not inserted during AAV HR. The substrate for AAV-mediated HR is the single stranded AAV genome. Double-stranded AAV genomes do not undergo HR (Vasileva et al., 2006). In addition, long regions of homology are required for successful alignment of the editing vector and the target chromosomal locus. The presence of homology arms is critical for alignment with the chromosomal genomic sequence followed by crossover and resolution of the crossover junction within the homology regions at the 5′ and 3′ ends. Since the crossovers occur within the homology arms, the ITRs are excluded and not inserted into the chromosomal locus following AAV-mediated HR.

However, in the absence of homology arms, any double-stranded or self-complementary AAV DNA in the nuclei appear to be dropped into sites of the DSB regardless of whether they are created by nucleases associated with editing platforms or created by environmental conditions such as irradiation and chemicals. For random integration of AAV and in most nuclease-based platforms, the insertion of double-stranded AAV genomes into the sites of chromosomal DSBs results in the insertion of ITRs, often in rearranged forms.

## Setting the Cellular Stage for AAV Gene Editing

AAV vectors specifically designed for genome editing in the absence of programmable nucleases function in a distinctly different manner from either WT AAV or Rep-free AAV gene transfer vectors. Infection of cells with single-stranded AAVs initiates a cellular DNA damage response (DDR), although no accompanying actual cellular DNA damage has been identified (Jurvansuu et al., 2005; Choi et al., 2006; Jurvansuu et al., 2007; Cervelli et al., 2008; Ingemarsdotter et al., 2010; Hirsch, 2015). The initiation of DDR in target cells likely provides an ideal environment for genome editing by AAV. It is possible that the single-stranded AAV genome with the structured ITRs resembles a stalled replication fork and therefore elicits a DDR in infected cells. AAV infection has been shown to cause cell cycle arrest, primarily in the G1 phase of the cell cycle associated with activation of checkpoint proteins ATR, Chk1, and H2AX (Jurvansuu et al., 2007; Fragkos et al., 2009). In cells bearing mutations of certain DNA repair proteins or p53, this AAV-induced DDR leads to catastrophic mitosis and cell death. In normal cells, however, no deleterious effects have been observed. The mechanism by which the cell cycle arrest is relieved in normal cells remains unknown. DDR activation in AAV infected cells likely contributes to the onset of HR if other conditions are met.

Increased targeted integration by AAVs was originally attributed to the increased availability of vector genomes in the nucleus, increased stability of AAV vector genomes due to the structured ITRs, and the potential of single-stranded genomes to participate in HR (Figure 2D). The hairpin structures formed by ITRs at the ends of the AAV genome likely prevent degradation of the single-stranded AAV genomes by nucleases, increasing their stability and availability for genome targeting in the presence of flanking homology arms. Similar findings have been reported in yeast and fungal species where it was observed that the linear plasmids with telomeres or terminal palindromic repeats were more stable in episomal configurations compared to those without telomeric ends. These were additionally found to be capable of integration if they contained regions of homology to the genomic sequence (Bijlani et al., 2019). It is likely that the structure of ITR and the junction with the single-stranded AAV genome is identified as stalled replication fork and initiates DDR resulting in the recruitment of DNA repair proteins (Shechter et al., 2004; Smith et al., 2018). The efficiency of HR-based gene targeting by AAVs may depend upon the design of the editing vectors and transduction conditions. While not absolute, improved editing efficiency was associated with an increase in multiplicity of infection (MOI), the use of longer homology arms, and central positioning of the insert sequences. However, in some studies, the use of asymmetric homology arms appeared to be more efficient in editing certain genes (Russell and Hirata, 1998; Hirata and Russell, 2000; Gaj et al., 2016; Smith et al., 2018). In some, but not all cases, it was found that editing increased over time, possibly due to delayed transduction of target cells or slow nuclear accumulation or uncoating of AAV, possibly correlated with cell cycle progression (Russell et al., 1994) and AAV capsid serotype (Smith et al., 2018). It has been reported that transcriptionally active sites and targeting loci transcribed in the opposite direction of the gene-targeting event may be easier to edit (Deyle et al., 2014; Spector et al., 2021). Genomic sequence and potential secondary structure of the target sites have also been shown to play important roles in guiding editing efficiencies. In general, however, it has been difficult to define a set of universal rules that would uniformly apply to AAV editing at all genomic loci. As of now, optimal editing constructs must be empirically defined for different genomic target loci. A broad range of genome modifications have been achieved using AAV-based genome engineering. These include introduction of nucleotide substitutions, deletions, and insertions at multiple loci in the genome.

Notably, genome editing by AAV does not require the addition of exogenous nucleases and likely utilizes a highly precise and probably non-canonical HR pathway (Figures 1 and 2D). However, there are specific critical requirements for AAV-based editing vectors. AAV editing vectors must include: 1) the presence of ITR sequences, 2) a single-stranded AAV vector genome, and 3) the presence of homology arms complementary to the target genomic sequences. Self-complementary and dimeric AAV genomes do not edit, and thus, the single-stranded configuration of AAV vectors is essential for gene editing (Hirata and Russell, 2000).

## AAVHSC Editing Vectors

We have recently shown that AAV editing vectors belonging to clade F mediate exclusively HR-based, highly accurate and seamless, and efficient genome editing in human cells and mice without the requirement for exogenous nucleases (Figure 2D) (Smith et al., 2018). These vectors include a family of novel, naturally occurring AAVs isolated from healthy human hematopoietic stem cells (HSCs), termed AAVHSC (Smith et al., 2014; Chatterjee et al., 2020). AAVHSC editing was found to be precise and efficient, with no on-target insertion/deletion mutations and no evidence of genomic scarring or incorporation of residual viral sequences, including the ITRs. A variety of human cell types, including human CD34^+^ hematopoietic stem cells, liver sections, hepatic sinusoidal endothelial cells, myoblasts, and immortalized human B lymphoblastoid cell lines, were successfully edited using AAVHSCs. We reported a median editing efficiency of 24.2% for clade F AAVs, with some serotypes showing up to 50% efficiency. In comparison, the editing efficiency of clade B AAV was 2.12%, and clade E AAV was 1.7%. The reasons underlying the increased efficiency of certain AAV serotypes remain unknown.

Interestingly, we observed efficient editing of post-mitotic tissues *in vivo*. Sequence analyses again revealed high on-target precision with no indels or ITR insertions observed. *In vivo* targeted insertion of the promoterless luciferase open reading frame into intron one of the Rosa26 locus resulted in long-term luciferase expression, indicating that AAVHSCs were capable of efficient and stable *in vivo* genome editing. In addition to targeted insertion, AAVHSC editing vectors also mediated nucleotide substitutions with high accuracy. In another study, an AAVHSC15 editing vector was used to edit the PAH gene on chromosome 12, mutations of which result in phenylketonuria. The editing vector targeted the PAH cDNA to intron one of the PAH gene. *In vivo* editing in a chimeric human liver xenograft model resulted in editing 6% of human hepatocytes. No on-target mutations were found following NGS sequencing spanning both homology arms (Chen et al., 2020). Thus, AAVHSCs represent a promising platform for seamless, high-efficiency gene editing and corroborate the use of AAVs as a genome engineering tool.

## Mechanism of AAV Editing

Although the mechanism of AAV gene targeting has yet to be definitively delineated, we have recently shown that AAV editing requires homology to targeted chromosomal sequence, single-stranded AAV editing genomes, and the presence of BRCA2 in target cells (Smith et al., 2018) (Figure 1). In the absence of BRCA2, no editing was observed by any AAV serotype. Based upon the requirements for homology arms and BRCA2, our results strongly suggest that AAVs utilize the HR pathway to carry out editing (Vasileva and Jessberger, 2005; Vasileva et al., 2006; Smith et al., 2018). AAV is the only currently used genome editing platform that does not require the prior creation of double-stranded DNA breaks by exogenous nucleases and appears to exclusively use the high-fidelity HR pathway to mediate genome editing.

## Single-Stranded AAV Genomes Are Necessary for Editing

Only single-stranded AAV genomes can mediate editing, and self-complementary or double-stranded AAV genomes do not function in this capacity (Hirata and Russell, 2000). This requirement for single-stranded AAV genomes most likely reflects their role in annealing to homologous genomic sequences. We examined the genome forms of the editing vectors in the murine liver 6 months after intravenous injection of editing vectors (Smith et al., 2018). Notably, we did not detect either free monomeric or circular concatemeric vector genomes in the liver, suggesting that the editing vector genomes may not have initiated second-strand synthesis or did not form double-stranded concatamers commonly observed following AAV transduction (Smith et al., 2018). Upon nuclear entry and uncoating, single-stranded AAV genomes undergo second-strand synthesis and form circular concatamers (Figure 2A). It is possible that a prolonged single-stranded phase of editing vector genomes may enhance editing efficiencies. Thus, AAV serotypes that promote extended survival of single-stranded genomes may be more proficient at mediating HR.

## Role of the Structured AAV ITR in Initiating HR

Based on our observation that editing by every AAV serotype was abolished in the absence of BRCA2 (Smith et al., 2018), we concluded that BRCA2 plays a critical role in AAV editing and that there is no redundancy provided by any other alternate repair protein. We hypothesized that BRCA2 recognizes the double-strand to single strand junction at the ends of the ITRs, then recruits RAD51, displacing RPA from the coated single-stranded AAV genome and forms the nucleofilament necessary to initiate HR (Figure 1). This likely leads to the assembly of the HR complex, including BRCA1, PALB2, and Rad51 (Figure 1).

We previously reported that a reduction in editing efficiencies was observed with all AAV serotypes in cell lines bearing homozygous or compound heterozygous mutations in BLM helicase, FANCA, FANCC, FANCD2, and FANCF, indicating their possible involvement in AAV-mediated HR (Smith et al., 2018). However, another study showed that knockout of FANCM when combined with a knockout of BLM or RMI1 increased AAV HR (de Alencastro et al., 2021). Whether these differences in the HR-related outcomes were a result of the interaction of these proteins or were unique to the limited number of clones studied is unclear. ATR may also play a role in DNA repair upon recognition of the single-stranded AAV genome structure resembling a stalled replication fork. ATR in association with ATR interacting protein (ATRIP) is known to signal cell cycle checkpoint activation in the presence of stalled replication forks and single-stranded DNA gaps (Shechter et al., 2004). It is likely that some redundancy in the proteins involved accounted for the persistence of some editing activity in these cells. This is supported by reports of ATR colocalizing with the AAV genome in nuclear foci in the absence of ATM and NBS1 (Jurvansuu et al., 2005). Redundancy is known to exist for the proteins of the HR complex and is thought to be evolutionarily important for the maintenance of genomic integrity.

We observed little or no change in HR in cells bearing homozygous or compound heterozygous mutations in FANCB, ATM, NBS1, ERCC4/XPF, and RAG1. This was in contrast to the complete abolition of AAV HR in cell lines with compound heterozygous mutations in BRCA2 and a reduction of HR in cells with mutations in BLM helicase, FANCA, FANCC, FANCD2, and FANCF (Smith et al., 2018). Evaluation of the roles of these proteins in AAV editing will further clarify the mechanisms involved in AAV editing and any built-in redundancies in the involved pathways.

In addition to HR, AAVs may additionally use a non-canonical HR pathway. It has been shown that silencing of the NHEJ protein DNA-PK did not affect gene targeting efficiency; however, silencing of HR proteins, RAD54L or RAD54B, and partial silencing of the RAD51 paralogue XRCC3 reduced or abolished AAV gene targeting (Vasileva et al., 2006). RAD54L and RAD54B belong to the RAD52 epistasis group, and RAD54L facilitates strand exchange by RAD51 (Kanaar et al., 1996; Hiramoto et al., 1999; Sigurdsson et al., 2002). RAD51 replaces RPA on single-stranded DNA, forming a nucleoprotein filament (Jensen et al., 2010). XRCC3 binds to ssDNA along with RAD51C and slows down the replication fork progression upon DNA damage (Masson et al., 2001; Henry-Mowatt et al., 2003). Recently XRCC3 was shown to bind to DNA directly, promoting nucleoprotein formation by RAD51 (Forget et al., 2004). Thus, the involvement of HR proteins RAD54, RAD51, and BRCA2 suggests that the single-stranded AAV genome structure may indeed be recognized as a stalled replication fork, which sends a signal to BRCA2 (Figure 1). BRCA2 then recruits RAD51 and its paralogues to the site and stimulates RAD51-mediated nucleoprotein filament formation resulting in HR-mediated gene targeting.

The MRN (MRE11, RAD50, NBS1) complex associates with AAV vector genomes and limits the conversion of the single-stranded AAV genome to the double-stranded form (Sanlioglu et al., 2000; Cervelli et al., 2008). For AAV gene transfer vectors, MRN and ATM reduce transduction efficiency by preventing second strand synthesis resulting in reduced transgene transcription. However, for gene editing, recognition of the structured AAV genome and the subsequent binding by MRN may stimulate HR by inducing ATM in the presence of homologous sequences. This further underscores the lack of editing by self-complementary AAV genomes as single and double-stranded junctions are not available for assembly of the HR complex. The association of RAD52 and the MRN complex with AAV ITRs has also been reported (Zentilin et al., 2001; Schwartz et al., 2007). It was hypothesized that RAD52 might be involved in the replication of AAV via break-induced replication (BIR), where replication proceeds unidirectionally, resulting in a formation of double-stranded intermediate (Hendrickson, 2020). It is possible that in the case of AAV vectors, the absence of AAV Rep proteins and limited double-strand synthesis by the MRN complex and binding to Rad52/MRN complex may further protect against nuclease-mediated degradation of the single-stranded AAV editing genome. Rad52 also mediates homology search (Rothenberg et al., 2008; Jalan et al., 2019). Thus, upon binding to the AAV editing genome, it may be instrumental in guiding the editing genome to the homologous region in the cellular genome.

## Role of the Cell Cycle in AAV HR

The HR mechanisms discussed above illustrate how AAVs may mediate gene targeting in dividing cells where HR occurs mainly during the S/G2 phases of the cell cycle. However, recent observations suggest that AAV also mediates genome editing in non-dividing HSCs, cardiomyocytes, and adult post-mitotic tissues *in vivo*, indicating that non-canonical HR pathways may be involved (Smith et al., 2018; Kohama et al., 2020). While little is known about how AAVs mediate HR in non-dividing cells, some common requirements appear to be critical. First, a high MOI of single-stranded AAV is essential to trigger HR *in vitro*. Furthermore, targeted insertion into cardiomyocytes was shown to utilize the Fanconi anemia pathway involved in single-strand break repair. Interestingly, an independent study uncovered a non-canonical HR pathway that used single-stranded donor templates to repair artificially generated single-stranded nicks at the target locus (Davis and Maizels, 2014). They further showed that this mechanism exhibited a much higher frequency of gene editing than the canonical HR, which required DSBs (Davis and Maizels, 2011). Whether AAVs interact with this pathway to mediate gene editing remains to be elucidated. If indeed they do, one critical missing link is the mechanism required to generate single-stranded breaks (SSBs). Do AAVs utilize random SSBs that occur at the target sites, or is it possible that SSBs are actively created at target sites? The probability of random SSBs occurring at a 2–4 kb homologous target site in the haploid genome is approximately one in a million. This is consistent with spontaneous targeting frequencies observed for plasmid DNAs (Porteus et al., 2003) but does not account for the significantly higher targeting frequency observed with AAV vectors. Therefore, it is likely that AAV editing may result in SSB generation at the target site. However, neither the AAV genome nor the capsid proteins have intrinsic nicking activity. It is possible that the AAV editing machinery recruits cellular nickases using the homology arms as a guide to direct the cleavage to a targeted genomic locus. Mre11 is a cellular nuclease that interacts with the AAV genome (Cervelli et al., 2008). Mre11 of the MRN complex plays a critical role in DNA repair by binding to DNA break sites and initiating resection through its exonuclease and endonuclease activities. As described above, several studies have shown that MRE11 mainly hinders AAV transduction by inhibiting second strand synthesis (Schwartz et al., 2007; Lentz and Samulski, 2015). It will be interesting to determine whether high MOIs of AAV editing vectors can alter MRE11 functions that may lead to the nicking of genomic DNA at target sites. Another possibility is that at high MOIs, single-stranded AAV genomes may hybridize to the homologous genomic DNA and activate DDR resulting in noncanonical HR. While there is little direct evidence supporting this model, recent studies may provide some hints.

## Transcription-Coupled HR

Another noncanonical HR pathway that is active in non-dividing cells is transcription-coupled HR (TC-HR), reported in yeast and mammalian cells (Keskin et al., 2014; Wei et al., 2015). Here, RNA-templated recombination repair mechanism was identified in the G0/G1 cell cycle phase, mediated by the Cockayne syndrome B protein (CSB), RPA, RAD51, RAD51C, and RAD52 at the site of DNA breaks (Wei et al., 2015). This process was shown to be initiated by CSB, which further recruited the HR proteins to the site. TC-HR also contributed to DNA repair in post-mitotic neurons (Welty et al., 2018), with the recruitment of RAD52 to the site of DNA breaks. A gene targeting study conducted with AAVs concluded that transcriptionally active sites exhibited improved AAV-mediated HR (Spector et al., 2021). A study conducted in yeast reported the formation of Rad51 foci on DSBs in the G1 phase of the cell cycle in the absence of Rad52 foci, which is required for Rad51 recruitment (Smith et al., 2019). It was hypothesized that Rad52 may be present in low abundance, albeit at sufficient levels to recruit Rad51, but not enough to form Rad52 foci. Other factors may regulate HR during the G1 phase of the cell cycle. For instance, some histone modifications associated with transcription are also known to influence the recruitment of DNA repair proteins. For example, H3K36me3, a histone marker of transcription elongation, is known to recruit CtIP and RAD51 at transcriptionally active sites (Aymard et al., 2014), and TIP60-dependent H4 acetylation recruits BRCA1 to the sites of DSBs (Tang et al., 2013), which suggests possible regulation of HR during transcription. Histone marks H4K20me1 and H4K20me2 present in euchromatin regions that undergo active transcription recruit repair factor p53 binding protein1 (53BP1) to DSBs that plays a role in NHEJ (Botuyan et al., 2006). Thus, there is a balance between the HR and NHEJ repair pathways, and one potential regulator of the choice of the pathway appears to be histone modifications during transcription. This mechanism of TC-HR may also explain AAV-mediated HR in non-dividing cells where spontaneous breaks in the genome may occur during transcription rendering the genes accessible for editing.

It is well established that highly expressed genes commonly associate with R-loop formation where the RNA transcripts hybridize to the transcribed DNA strand resulting in the non-transcribed strand being exposed as single-stranded DNA (R-loop). This exposed section of single-stranded DNA is vulnerable to lesion formation that can result in DNA breaks (Crossley et al., 2019; Rinaldi et al., 2020). Since the R-loop structures formed during transcription are prone to damage (Aguilera and Garcia-Muse, 2012), it is possible that the damaged single-stranded genomic DNA in the R-loop structures hybridize to the single-stranded AAV genome and repair by HR ensues using AAV as the template. It has been shown that RAD52 binds to R-loop structures (Welty et al., 2018), which functions in homology search for repair. In such cases, the presence of a homologous sequence in the AAV editing vector genome in the vicinity of actively transcribing DNA could mediate targeted genome editing. Most lesions on one strand of DNA in the R-loop can be repaired by the classical nucleotide excision repair pathway (NER), which is not strictly dependent on cell division (Sollier et al., 2014; Marnef et al., 2017). Recently, an alternate repair mechanism was described that uses RNA transcripts instead of sister chromatids as repair templates and is therefore independent of cell division (Keskin et al., 2014; Meers et al., 2016; Welty et al., 2018; Ouyang et al., 2021). Both pathways are associated with transcription and are independent of the cell cycle. It would be interesting to investigate whether AAV vectors utilize these pathways to mediate gene editing.

## Genome Editing in the Clinic

Although the path from the bench to the clinic is long and arduous, clinical trials to test the safety and efficacy of therapeutic genome editing have been initiated. Therapeutic gene editing has focused upon genetic diseases and immuno-oncology (Mullard, 2020). Here we will focus primarily on gene editing for genetic diseases. Among the first editing platforms to enter the clinic were zinc finger nucleases and CRISPR/Cas9. ZFN and CRISPR/Cas9 have been used *ex vivo* to downregulate expression of CCR5, a receptor for HIV-1. Patients were infused with *ex vivo* edited CD4 cells. *Ex vivo* editing followed by infusion was found to be safe, although the level of edited cells was low in both studies (Tebas et al., 2014; Xu et al., 2019). One clinical trial tested the efficacy of *in vivo* systemic administration of ZFNs to treat mucopolysaccharidosis II (MPS II) (ClinicalTrials.gov Identifier: NCT03041324). AAV vectors encoding ZFNs targeted to the mutant iduronidase gene and a donor sequence encoding the wild type version were infused intravenously. Some modest benefits were noted, although they could not be definitively linked to the gene editing treatment due to limited sensitivity of the assays.

Hemoglobinopathies represent attractive targets for genome editing. Upregulation of fetal hemoglobin achieved by inactivation of BCL11A is an effective treatment for diseases caused by mutations in the beta globin gene. Two patients, one with transfusion dependent thalassemia and the other with sickle cell anemia were treated with autologous *ex vivo* CRISPR/Cas9 edited CD34^+^ hematopoietic stem cells. In both cases the BCL11A gene which suppresses fetal hemoglobin production was inactivated with CRISPR/Cas9. Following infusion of *ex vivo* edited cells, both patients expressed fetal hemoglobin. This resulted in the elimination of vaso-occlusive events and transfusion independence (Frangoul et al., 2021). Thus, while unanswered questions still remain, early results are encouraging.

In the first systemic *in vivo* trial of CRISPR/Cas9, lipid nanoparticles were used to deliver the mRNA for Cas9 and a guide RNA targeting transthyretin (TTR) (Gillmore et al., 2021). In this approach, the TTR gene was disabled by the introduction of a Cas9-mediated double stranded break in hepatocytes, resulting in a dose-dependent reduction in TTR expression. However, this approach targeted both the mutant and wild type TTR alleles and notably would be ineffective at reducing previously accumulated amyloid deposits. Nevertheless, promising early indications of efficacy were noted.

A clinical trial of CRISPR/Cas9 is underway to test subretinal delivery of an AAV5 vector encoding two guide RNAs and Cas9 designed to delete a mutation in the CEP290 gene associated with Leber’s Congenital Amaurosis (LCA), a common cause of childhood blindness (ClinicalTrials.gov Identifier: NCT03872479), (Maeder et al., 2019). Some efficacy was noted at the mid-dose levels, although adverse events including retinal tears, hemorrhage and inflammation were observed. (https://editasmedicine.gcs-web.com/static-files/22b32d3d-e38f-4e90-899c-a2e701872745), (Ledford, 2020).

Preclinical and investigational new drug (IND)-enabling studies in AAV-mediated therapeutic gene editing are being conducted by several groups. Notably, one study recently received IND clearance from the US Food and Drug Administration (FDA) for the evaluation of AAV-mediated gene editing for the treatment of phenylketonuria (PKU), an inborn error of metabolism caused by mutations in the phenylalanine hydroxylase gene, resulting in an inability to metabolize phenylalanine (https://www.homologymedicines.com/news-story/homology-medicines-announces-worlds-first-gene-editing-clinical-trial-for-pku). This trial will evaluate HMI-103, an AAVHSC15-based gene editing vector to edit the PAH gene and restore expression in PKU patients. While this is the first AAV genome editing study to enter the clinic, it will likely be followed by others to treat a spectrum of genetic diseases.

Although clinical gene editing is still in early stages, pioneering trials have generally yielded promising results. Long-term follow up of *ex vivo*, systemic and locally administered gene editing strategies using the diverse available platforms and delivery systems will be important to assess the overall safety and therapeutic efficacy and importantly, the potential impact of nuclease-mediated off target DNA breaks and the creation of indel mutations.

## Conclusion

AAV editing vectors represent an orthogonal editing platform that differs significantly from all current nuclease-based gene editing tools. It is the only genome editing platform that exclusively utilizes the high-fidelity cellular HR repair pathway and does not require exogenous nucleases for the a priori creation of DSBs. Both of these properties together lead to a highly precise editing outcome that preserves genomic integrity with no incorporation of indel mutations or viral ITR at the target site while also removing the possibility of genotoxicity associated with the creation of off-target DNA breaks and subsequent mutagenesis following error-prone repair.

All AAV serotypes possess the inherent ability to mediate accurate editing of cellular genomes *in vitro* and *in vivo*. AAV-based editing incorporates the key hallmarks of HR, including the absolute requirement for BRCA2 and homology between the repair template and the genomic target. Notably, however, unlike classical HR, AAV editing is not restricted to dividing cells, and post-mitotic cells *in vivo* undergo efficient editing by AAVs. While all AAV serotypes mediate genome editing, specific serotypes display increased editing efficiency, offering the prospect of effective *in vivo* editing. AAV editing vectors also serve as their own *in vivo* delivery vehicles that have been proven safe in numerous gene therapy trials. Some AAV vectors also possess the ability to cross the blood-brain barrier and transduce cells of the central nervous system (CNS), thus potentially enabling the much-needed ability to repair mutations in the CNS. Further insights into the mechanism of how AAV co-opts the cellular HR pathway to carry out high-fidelity editing and how some AAV serotypes amplify the efficiency of HR will be necessary for the further evolution of genome editing for the permanent correction of inherited and acquired genetic diseases.
